# An ontology for microbial phenotypes

**DOI:** 10.1186/s12866-014-0294-3

**Published:** 2014-11-30

**Authors:** Marcus C Chibucos, Adrienne E Zweifel, Jonathan C Herrera, William Meza, Shabnam Eslamfam, Peter Uetz, Deborah A Siegele, James C Hu, Michelle G Giglio

**Affiliations:** Institute for Genome Sciences and Department of Microbiology and Immunology, University of Maryland School of Medicine, Baltimore, MD USA; Department of Biochemistry and Biophysics, Texas A&M University and Texas AgriLife Research, College Station, TX USA; Current address: University of California, San Francisco, CA USA; Department of Biology, Texas A&M University, College Station, TX USA; Virginia Commonwealth University, Richmond, VA USA; Institute for Genome Sciences and Department of Medicine, University of Maryland School of Medicine, Baltimore, MD USA

**Keywords:** Annotation capture, Bacterial phenotype, Biological ontologies, Controlled vocabulary, *Escherichia coli*, Microbial genetics, Phenotype annotation, Phenotypic evidence, Wiki

## Abstract

**Background:**

Phenotypic data are routinely used to elucidate gene function in organisms amenable to genetic manipulation. However, previous to this work, there was no generalizable system in place for the structured storage and retrieval of phenotypic information for bacteria.

**Results:**

The Ontology of Microbial Phenotypes (OMP) has been created to standardize the capture of such phenotypic information from microbes. OMP has been built on the foundations of the Basic Formal Ontology and the Phenotype and Trait Ontology. Terms have logical definitions that can facilitate computational searching of phenotypes and their associated genes. OMP can be accessed via a wiki page as well as downloaded from SourceForge. Initial annotations with OMP are being made for *Escherichia coli* using a wiki-based annotation capture system. New OMP terms are being concurrently developed as annotation proceeds.

**Conclusions:**

We anticipate that diverse groups studying microbial genetics and associated phenotypes will employ OMP for standardizing microbial phenotype annotation, much as the Gene Ontology has standardized gene product annotation. The resulting OMP resource and associated annotations will facilitate prediction of phenotypes for unknown genes and result in new experimental characterization of phenotypes and functions.

## Background

Phenotypes are the observable characteristics of an organism that result from the expression of a particular genotype in a particular environment. Traditionally, phenotypes and genotypes were linked together by studying a mutant phenotype of interest followed by genetic mapping and characterization to identify the genetic change that is responsible for the phenotype, a process known as forward genetics. Recently, high-throughput reverse genetics methods are increasingly being applied to observe phenotypic effects of targeted changes to genotypes with gene knockouts [1] or changes to gene expression patterns with methods like RNA interference [2]. Such studies have identified thousands of phenotype-genotype associations.

Until recently, microbial phenotypic information has been largely captured as free text descriptions in primary research papers, review articles, and compilations such as genetic maps or *Bergey’s Manual of Determinative Microbiology* [3]. Unfortunately, ambiguities in natural language confound attempts to retrieve similar information across data sources. For example, “serotype” or “serovar” both refer to the same phenotypic trait, but a simple text-based computer query with either word alone would miss the other. Likewise, one term can be ambiguous: “sporulation” can be used to describe the general process of spore formation, but it is usually used to refer to a more precise concept such as sporulation to survive adverse conditions (such as endospore formation in *Bacillus*) or sporulation for the purpose of reproduction (such as found in *Actinomyces*). A search just for “sporulation” could return imprecise results. Issues such as these hamper the ability to integrate different phenotypic data sets for the same organism or to leverage known phenotypic information in one strain/species to predict possible phenotypes in other strains/species. Ideally, phenotype information should be stored in a consistent, computable format for ease of data integration and mining.

**Figure 1:**
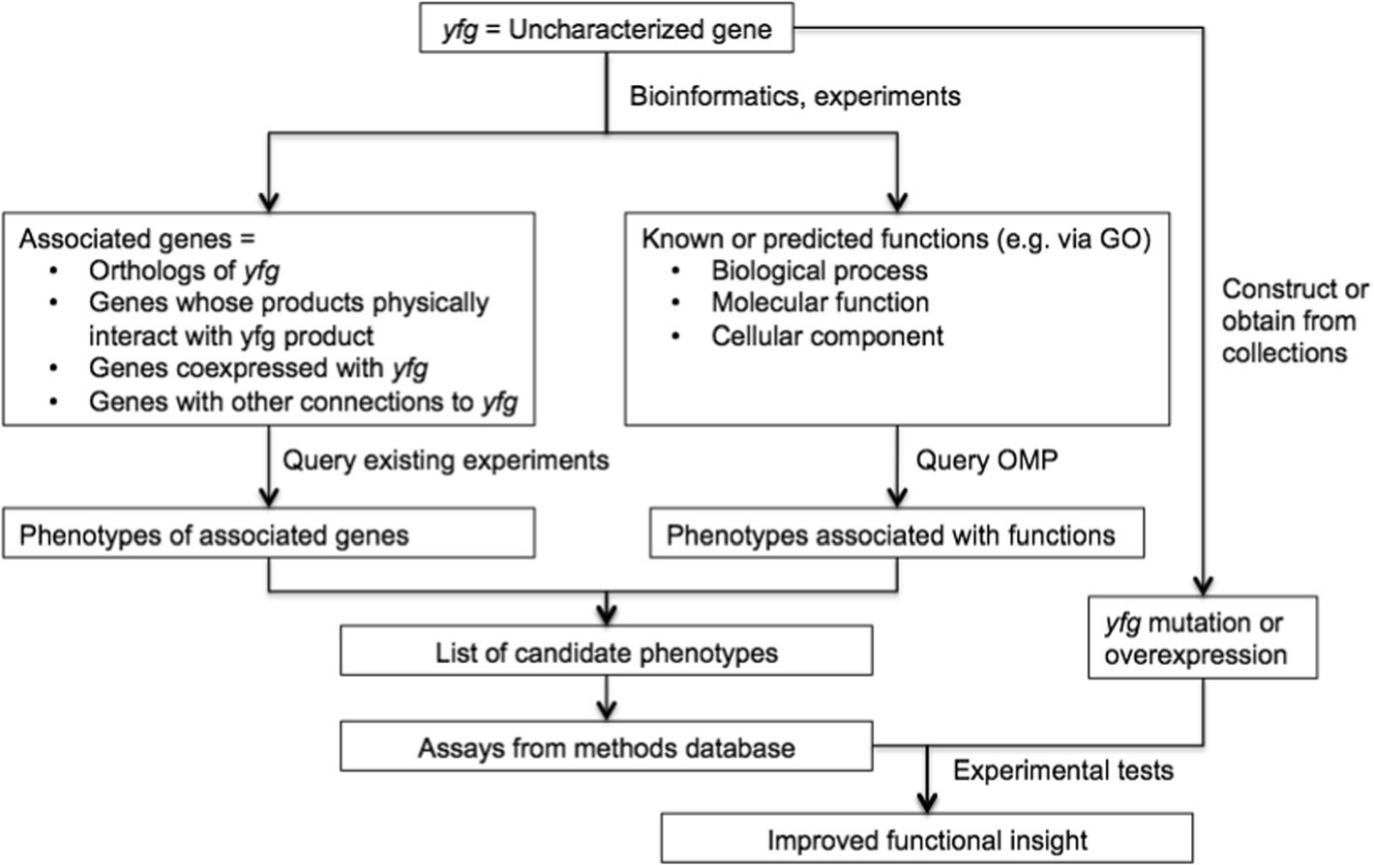
**Using a phenotype ontology to elucidate gene function.**

Controlled vocabularies, including ontologies, are commonly used to provide both consistent terminology and a structured data format for the capture of biological information [4]. An ontology consists of a controlled vocabulary of defined terms with unique identifiers and precise relationships to each other. In such a system, synonymous concepts are all encapsulated (as synonyms) within a primary term with a single identifier. In cases where the same word refers to multiple concepts, each concept is made into a separate term with a unique identifier and a definition that provides information on the precise meaning, and each term is placed in an appropriate area of the ontology in relation to other terms. When using an ontology for capturing phenotype information, terms describing each phenotype are linked to particular genotypes through the process of annotation. Thus, effective query and comparison of phenotype data across multiple datasets, organisms, and strains can be accomplished. Such a dataset could have major applications in functional phenomics, the elucidation of gene function from phenotypes (Figure 1). Uncharacterized genes could be linked to genes that are associated with known phenotypes using a number of bioinformatics approaches including orthology, genomic context (such as operons), co-expression, and protein-protein interactions [5-7]. This would provide a set of predicted phenotypes for the uncharacterized genes that can be tested. A second application for such a dataset would be that of strain variant analysis. Increasingly, multiple strains of one species (up to several hundred or thousand) are being sequenced. These strains often vary significantly in both gene content and phenotypic characteristics, including for example host range, antibiotic resistance profiles, and virulence. The ability to correlate differences in gene content with phenotypic differences could help target genes likely to be involved in these processes and advance research into infectious disease and human health [8].

Currently, there are anatomy and phenotype ontologies in common use for many multicellular organisms including mouse [9], *Drosophila* [10], *Caenorhabditis elegans* [11], zebrafish [12], and plants [13], enabling improved analysis of phenotype data. Similar efforts for microbes include ontologies for *Schizosaccharomyces pombe* [14] and Ascomycetes [15]. However, none of the existing ontologies is appropriate to comprehensively capture phenotypes for Bacteria or Archaea or for enabling comparisons across microbial species. Here we describe the development of a general Ontology of Microbial Phenotypes (OMP)*.* Initial term development efforts have been focused on bacterial phenotypes. However, future OMP development will expand the scope to include Archaea and eventually all microbes. OMP can be accessed via a wiki-based ontology browser [16] and from the SourceForge development site [17].

## Implementation

### OMP structure and organization

OMP is a general microbial phenotype ontology applicable to diverse microbes. Microbial phenotypes include properties of single organisms as well as populations of cells, such as colonies, biofilms, pellicles, and liquid cultures. Entities described by OMP include phenotypes that are directly observed and phenotypes that are inferred from indirect evidence. Examples of directly observed phenotypes are cell and colony morphologies and the presence or absence of motility. Inferred phenotypes include most physiological phenotypes. For example, while it is technically possible to observe utilization of a sugar by isotopic tracer experiments, it is much more common to infer that utilization is present or absent based on the ability or inability of the sugar to support growth or transform indicator dyes.

Furthermore, there are two distinct classes of phenotypes: independent phenotypes that provide information about an organism without reference to any other information and dependent phenotypes that capture a relative difference observed when comparing (at least) two separate genotypes or conditions. To illustrate the difference between independent and dependent phenotypes consider the phenotypes associated with motility. If a strain of *E. coli* were observed to move, it would have the independent phenotype “presence of cell motility”. However, if a mutation in a particular *E. coli* strain led to a reduction of motility, then that strain would be described by the dependent phenotype “decreased cell motility,” which is relative to the strain used as a reference. Organisms that are non-motile, such as *Klebsiella pneumonia*, would have the independent phenotype “absence of cell motility” [3]. Terms for both independent and dependent phenotypes are essential to represent the richness of genetics and genetic interactions (for examples, see [18-22]). OMP and its associated annotation system have been designed to capture all of these types of phenotypes. In the ontology, terms for independent and dependent phenotypes can be distinguished by their definitions as well as by the fact that dependent phenotypes are linked to relevant parent class “altered phenotype” terms. We have examined the term structures of related ontologies such as the Fission Yeast Phenotype Ontology (FYPO) [14] in order to ensure compatibility between OMP and other resources. We hope to ultimately develop OMP to be able to represent all phenotypes in other ontologies related to microbes allowing analysis of annotations to be linked together into one system.

**Figure 2:**
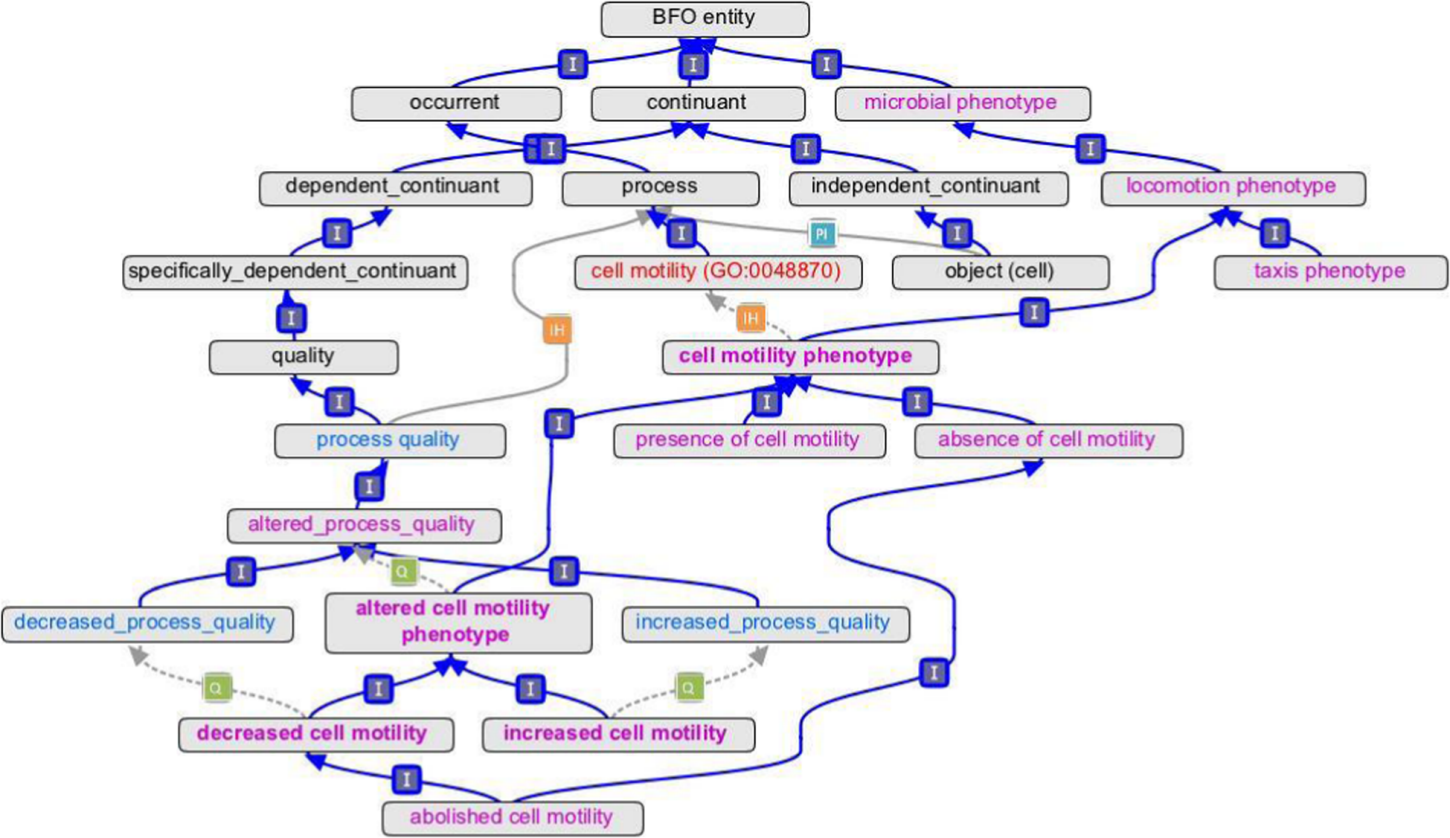
**Ontology of Microbial Phenotypes (OMP) in the context of Basic Formal Ontology (BFO), Phenotypic Quality Ontology (PATO), and Gene Ontology (GO).** Terms from respective ontologies are rendered in different color type: BFO, black; PATO, blue; GO, red; and OMP, purple. (Note that “quality” exists in both BFO and PATO, and PATO instantiates the concept of “process quality”.) Asserted relationships are indicated by solid lines, and relationships inferred by a reasoner are indicated by dotted lines. Abbreviations: I, is_a; IH, inheres_in; PI, participates_in; Q, has_quality.

OMP is built using foundations provided by Basic Formal Ontology (BFO) [23] and Phenotype and Trait Ontology (PATO) [24]. BFO provides a framework of upper level terms to support domain-specific ontology development. PATO contains phenotypic attributes for use in developing phenotype terms. Figure 2 shows how OMP relates to BFO and PATO, using terms related to motility as an example. All OMP terms (classes) descend from a root class called “microbial phenotype”. Because microbial phenotypes describe both processes (e.g. motility) and objects (e.g. cells), the OMP root class would be a direct child of the BFO root class “BFO entity.” However, the more granular (specific) OMP classes also connect to either the “continuant” or “occurrent” branches of BFO [25] by way of external ontologies, such as PATO or GO, as shown in Figure 2. Continuants are entities that persist through time, and include both objects, such as a bacterial cell or colony, and qualities of objects, such as cell shape or colony color. Occurrents are entities that have a temporal part and that occur through time, and include processes that an object can participate in, for example cell motility. In order to qualify processes (e.g. “decreased cell motility”), OMP follows the model of PATO, which defines “process quality” (PATO:0001236) as “a quality which inheres in a process.”

**Figure 3:**
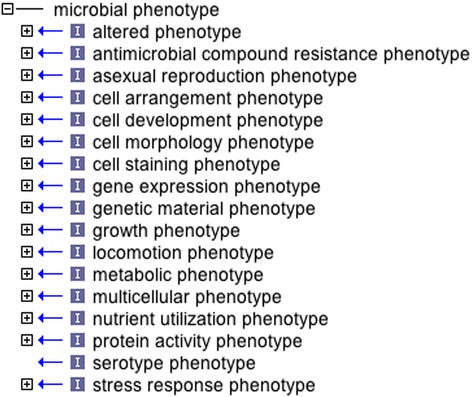
**Root class and high-level grouping terms of the Ontology of Microbial Phenotypes.**

We have included umbrella terms to group independent and dependent phenotype terms that refer to the same biological concepts (Figure 3). For example, the terms “presence of cell motility”, “absence of cell motility”, and “decreased cell motility” are all related to the same biological concept (cell motility) and are all thus also subclasses of “cell motility phenotype”, itself a subclass of “locomotion phenotype” (Figure 2). “The umbrella term names end in the word ‘phenotype’ (Figure 3).” These terms are particularly useful for querying of related annotations. We recommend that they not be used directly in annotations as there will usually be a more specific child term available. These terms are similar to the Gene Ontology (GO) grouping terms such as “cell part”, GO:0044464, which GO guidelines indicate should not be used for annotation [26,27].

Finally, OMP employs the qualifier “altered_relative_to” for dependent phenotype terms, which denote a difference in phenotype between organisms of two different genotypes or the same genotype assessed in two different environments. We have avoided the use of the qualifier “abnormal” for two reasons: there can be no consistent definition of what is “normal” or “abnormal” across organisms, and “altered_relative_to” is superior for capturing phenotypes of genetic modifiers such as suppressors and enhancers [28]. An altered phenotype is always relative to the phenotype of a designated control, which can be specified in the annotation (see below), or set to an arbitrary reference within an annotation set (e.g. a wild-type reference genotype within a species).

### Term development

All terms in OMP have genus-differentia definitions, where a term is a subclass of a parent term and is distinguished from other related subclass terms (siblings) by some differentiating characteristic unique to that term (and could be phrased as “B is an A that C’s”). For example, “cell motility by gliding” (B) is a “presence of cell motility phenotype” (A) that has the differentia “where a cell or cells exhibits smooth movement along a solid surface” (C). Terms also contain synonyms that allow for more effective keyword searches to be conducted. For instance, the OMP term “serotype” has synonyms “antigenic typing phenotype,” “serological test phenotype,” and “serovar.”

In addition to standard definitions and synonyms, many OMP terms contain a cross product, a special type of logical definition that represents the intersection of two or more terms [29]. These are based on a specified genus and a collection of differentiae. OMP cross products are derived by using relationships to connect a term from within the ontology to terms from external ontologies. For example, the OMP term “motility phenotype” is equivalent to the intersection of the OMP root term “microbial phenotype” (OMP:0000000) and the GO biological process term “cell motility” (GO:0048870) using the OBO Relations Ontology [30] relationship “inheres_in” (RO:0000052). In OBO syntax, this logical definition is expressed as:

- OMP:0000001
- name: cell motility phenotype
- is_a: OMP:0000312 ! locomotion phenotype
- intersection_of: OMP:0000000 ! microbial phenotype
- intersection_of: inheres_in GO:0048870 ! cell motility
- intersection_of: inheres_in GO:0005623 ! cell

Cross products are also used in OMP to implement the entity-quality (E-Q) method of phenotypic character description. In the E-Q model a bearer entity (e.g. a cell, cell part, or biological process) is associated with a quality (e.g. shape, decreased number, or altered duration), and together these comprise the “phenotype” [31]. OMP uses entity terms from OMP or other appropriate ontologies such as the GO along with qualities from PATO. For example, the term “decreased cell motility” is a type of “altered cell motility,” with the added quality “decreased process quality.” In this example, “altered cell motility” comes from within OMP and “decreased process quality” comes from PATO. The two terms are joined by the relationship has_quality (RO:0000086). In OBO syntax, this logical definition takes the form:

- OMP:0000002
- name: decreased cell motility
- is_a: OMP:0007001 ! altered cell motility
- intersection_of: OMP:0007001 ! altered cell motility
- intersection_of: has_quality PATO:0002302 ! decreased process quality

Whenever OMP term logical definitions comprise terms from external ontologies, OMP policy is to work with those developers to achieve interoperability.

Most of the existing OMP terms were generated as the result of OMP working group meetings held at Texas A&M University (College Station, TX), the Institute for Genome Sciences (Baltimore, MD), or by conference call. The term generation process includes examination of texts such as *Bergey's Manual of Determinative Microbiology* [3] and microbial literature, particularly genetic papers and reviews that are enriched for phenotype information (e.g. [1,22,32,33]). The process of establishing the structure of OMP and building the initial term set was carried out over several months during the beginning stages of the project. Since then, we have periodically refined the structure, as needed based on feedback from the community and project annotators. Now that annotation using OMP has begun in earnest (see more information below) we are able to engage in annotation-driven term development in which annotators identify phenotypes of interest from publications and request new terms as needed. This allows us to ensure that the ontology contains the terms and structure needed to fully capture real-world information as it will be encountered by annotators outside of our group. Ongoing development of OMP is in response to OMP literature curators, focusing on the genetic literature of the well-studied model organism, *E. coli* K-12. Following the model of the Gene Ontology [26] and other National Center for Biomedical Ontology ontologies [34], OMP is designed to support community participation in term development through the use of a public tracker [35].

OMP is developed in Open Biological Ontologies (OBO) format syntax with the ontology editor OBO-Edit [36]. A term tracker [35] is used to manage term requests, which are researched and added into the ontology. The built-in OBO-Edit reasoner is run to check term relationships and validity of cross-product terms.

## Results and discussion

### Accessing OMP

The first version of OMP was released in June 2011 on the OMP project’s development site [17], and new versions are released on a regular basis. Both a developer’s version and official release versions of OMP can be viewed at or downloaded from the development site. As of this writing there are 724 terms in the OMP name space, of which 100% have definitions. We have also deployed a wiki [16] modeled on Gene Ontology Normal Usage Tracking System (GONUTS) [37] for exploring the ontology, and adding usage notes to terms. The preliminary annotations described above are publicly visible via the OMP wiki. This will be converted to a system for community contribution of phenotype annotations in the future. Users wishing to request additions or changes to OMP may do so using the issue tracker system at our development site [35].

### Use of OMP to capture phenotype annotations

**Figure 4:**
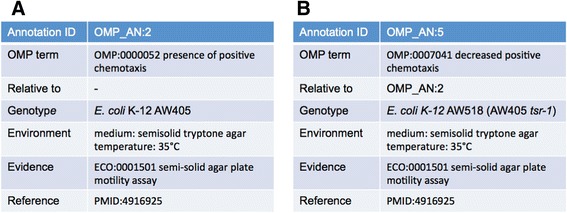
**Example annotations of chemotaxis phenotypes reported by Hazelbauer, et al. [** 40 **]. A)** an independent annotation showing chemotaxis in the parent strain based on a swimming assay in semisolid medium. **B)** a dependent annotation for a chemotaxis deficient mutant characterized by decreased swim diameter in the same soft agar assay. Decreased positive chemotaxis is relative to the genotype/environment combination specified in the annotation in **A**.

OMP can be used with any user-defined annotation system to capture phenotypes in a set of appropriately defined fields containing accessions or free text. OMP could be used in single-species annotation systems similar to the ones currently used at *Saccharomyces* Genome Database [38] and PomBase [39], where Yeast Phenotype Ontology and Fission Yeast Phenotype Ontology terms, respectively, are associated with gene records. However, to fully leverage the design of OMP we are developing an annotation data structure that will provide a more general and powerful mechanism to store both independent and dependent phenotypes associated with specific genes and alleles. A key element of this system will be identifiers associated with each annotation that will allow dependent phenotypes to be more fully expressed using relationships between annotations. Figure 4 shows two types of annotations: Figure 4A depicts an independent phenotype annotation while Figure 4B shows how a dependent annotation is made relative to the independent annotation in Figure 4A.

While the more complex annotation system is under development, we are continuing to collect phenotype information from publications on *E. coli* and storing the following elements: an identifier for the strain or genotype being annotated, a description of the environment or experimental conditions, an OMP term to capture the phenotype, an Evidence Ontology (ECO) term [7] to capture the evidence that supports the annotation, and the reference that was curated to make the annotation. So far we have associated phenotypes to observations made in 275 papers. These annotations connect OMP term development to the corpus of papers used to find examples of phenotypes that OMP must be able to support. Annotations can be viewed on the OMP wiki.

## Conclusions

To facilitate the effective capture and mining of phenotypic information for microbes we have undertaken development of an Ontology of Microbial Phenotypes. OMP consists of a controlled vocabulary and structured language where all terms are well-defined representations of microbial phenotypes. OMP is accessible through a centralized wiki-based data repository [16] and freely available for download [17]. Currently, OMP is being used to annotate phenotypes associated with *Escherichia coli* genes and strains.

The use of OMP with *Ec coli* will provide a model for other well-characterized bacterial systems such as *Salmonella* spp., *Bacillus subtilis* and *Caulobacter crescentus*. Annotation efforts will produce datasets for general use in bacterial ‘omic analyses including comparative and functional phenomics studies, genome annotation, microbiome studies, and research on infectious disease. Future work will focus on expansion of these resources to microbes beyond bacteria. Eukaryotic and prokaryotic microbes share many phenotypic traits, and we anticipate that these can all ultimately be captured in OMP allowing for even more powerful cross-species data mining and analysis.

## Availability and requirements

**Project name:** Ontology of Microbial Phenotypes

**Project home page**: Annotations & project wiki: http://microbialphenotypes.org. Open source project development site: http://purl.obolibrary.org/obo/omp/devel/. Issue tracker/term requests: http://purl.obolibrary.org/obo/omp/devel/omp-term-request. Latest stable ontology releases in OBO format: http://purl.obolibrary.org/obo/omp/omp.obo (full version) & http://purl.obolibrary.org/obo/omp/omp-simple.obo (version with no external terms).

**Operating system(s)**: Platform independent

**Programming language**: OBO format

**Other requirements**: OBO-Edit or other ontology viewer/editor is helpful to view the OBO file.

**License**: Creative Commons Attribution-ShareAlike 3.0 (CC BY-SA 3.0 US)

**Any restrictions to use by non-academics**: None

## Abbreviations

BFO: Basic formal ontology
ECO: Evidence ontology
OBO: Open biological ontologies
OMP: Ontology of microbial phenotypes
GO: Gene ontology
PATO: Phenotype and trait ontology
